# Locomotor Hyperactivity in the Early-Stage Alzheimer’s Disease-like Pathology of APP/PS1 Mice: Associated with Impaired Polarization of Astrocyte Aquaporin 4

**DOI:** 10.14336/AD.2022.0219

**Published:** 2022-10-01

**Authors:** Tianqi Wang, Yan Chen, Ying Zou, Yingting Pang, Xiaoxin He, Yali Chen, Yun Liu, Weixi Feng, Yanli Zhang, Qian Li, Jingping Shi, Fengfei Ding, Charles Marshall, Junying Gao, Ming Xiao

**Affiliations:** ^1^Jiangsu Province Key Laboratory of Neurodegeneration, Center for Global Health, Nanjing Medical University, Nanjing, 211166, China.; ^2^Brain Institute, the Affiliated Nanjing Brain Hospital of Nanjing Medical University, Nanjing, 210029, China.; ^3^Department of Human Anatomy and Histoembryology, Nanjing University of Chinese Medicine, Nanjing, 210023, China.; ^4^Department of Neurology, the Affiliated Nanjing Brain Hospital of Nanjing Medical University, Nanjing, 210029, China.; ^5^Department of Pharmacology, Shanghai Medical College, Fudan University, Shanghai, 200032, China.; ^6^College of Health Sciences, University of Kentucky Center of Excellence in Rural Health, Hazard, KY 41701, USA

**Keywords:** Alzheimer’s disease, amyloid-β, aquaporin 4, glymphatic system, hyperactivity

## Abstract

Non-cognitive behavioral and psychological symptoms often occur in Alzheimer's disease (AD) patients and mouse models, although the exact neuropathological mechanism remains elusive. Here, we report hyperactivity with significant inter-individual variability in 4-month-old APP/PS1 mice. Pathological analysis revealed that intraneuronal accumulation of amyloid-β (Aβ), c-Fos expression in glutamatergic neurons and activation of astrocytes were more evident in the frontal motor cortex of hyperactive APP/PS1 mice, compared to those with normal activity. Moreover, the hyperactive phenotype was associated with mislocalization of perivascular aquaporin 4 (AQP4) and glymphatic transport impairment. Deletion of the AQP4 gene increased hyperactivity, intraneuronal Aβ load and glutamatergic neuron activation, but did not influence working memory or anxiety-like behaviors of 4-month-old APP/PS1 mice. Together, these results demonstrate that AQP4 mislocalization or deficiency leads to increased intraneuronal Aβ load and neuronal hyperactivity in the motor cortex, which in turn causes locomotor over-activity during the early pathophysiology of APP/PS1 mice. Therefore, improving AQP4 mediated glymphatic clearance may offer a new strategy for early intervention of hyperactivity in the prodromal phase of AD.

Alzheimer's disease (AD) is the most common neurodegenerative disease. The disease not only adversely influences the health and life expectancy of patients, but also leads to negative emotions and mental distress to their relatives and caregivers [1, 2]. Unfortunately, there is no effective treatment for this devastating neurodegeneration currently [3]. Therefore, early diagnosis and discovery of new preventive measures are essential.

AD is accompanied by a variety of non-cognitive behavioral and psychological symptoms (BPSD), such as hyperactivity, disinhibition, impulsive behavior, apathy, hallucination, anxiety or depression [4-6]. In particular, hyperactivity and disinhibition often occur in prodromal AD patients [7-9], which may be associated with Amyloid beta (Aβ)-induced neuronal or network over-activation [10-12]. However, the mechanisms underlying these behavioral aberrations are largely unknown.

Aquaporin 4 (AQP4) is distinctively localized to astrocyte processes that are in direct contact with capillaries and pia, mediating glymphatic transport of macromolecules, including Aβ, out of the brain [13-15]. AQP4 is mislocalized on the neuron-facing membranes of activated astrocytes under a variety of pathological conditions, hampering clearance of Aβ and α-synuclein from brain parenchyma [16-18]. Recent studies have shown that an impairment of AQP4 polarization occurs prior to Aβ plague formation in several AD transgenic mouse models [19-22]. Deletion of AQP4 exacerbates AD-like pathology in APP/PS1 mice [23, 24]. Together, these data suggest that glymphatic dysfunction is a key step in the pathogenesis and progression of AD. However, an involvement of AQP4-mediated glymphatic transport in AD-related BPSD needs to be determined

In the present study, we have demonstrated that AQP4 mislocalization or deficiency increases intraneuronal Aβ load and neuronal activation in the motor cortex of APP/PS1 mice, thus showing a hyperactive phenotype in early-stage AD-like pathology. Improving glymphatic clearance of Aβ may serve as a promising target for early intervention for AD.

## MATERIALS AND METHODS

### Animals

**Figure 1.**
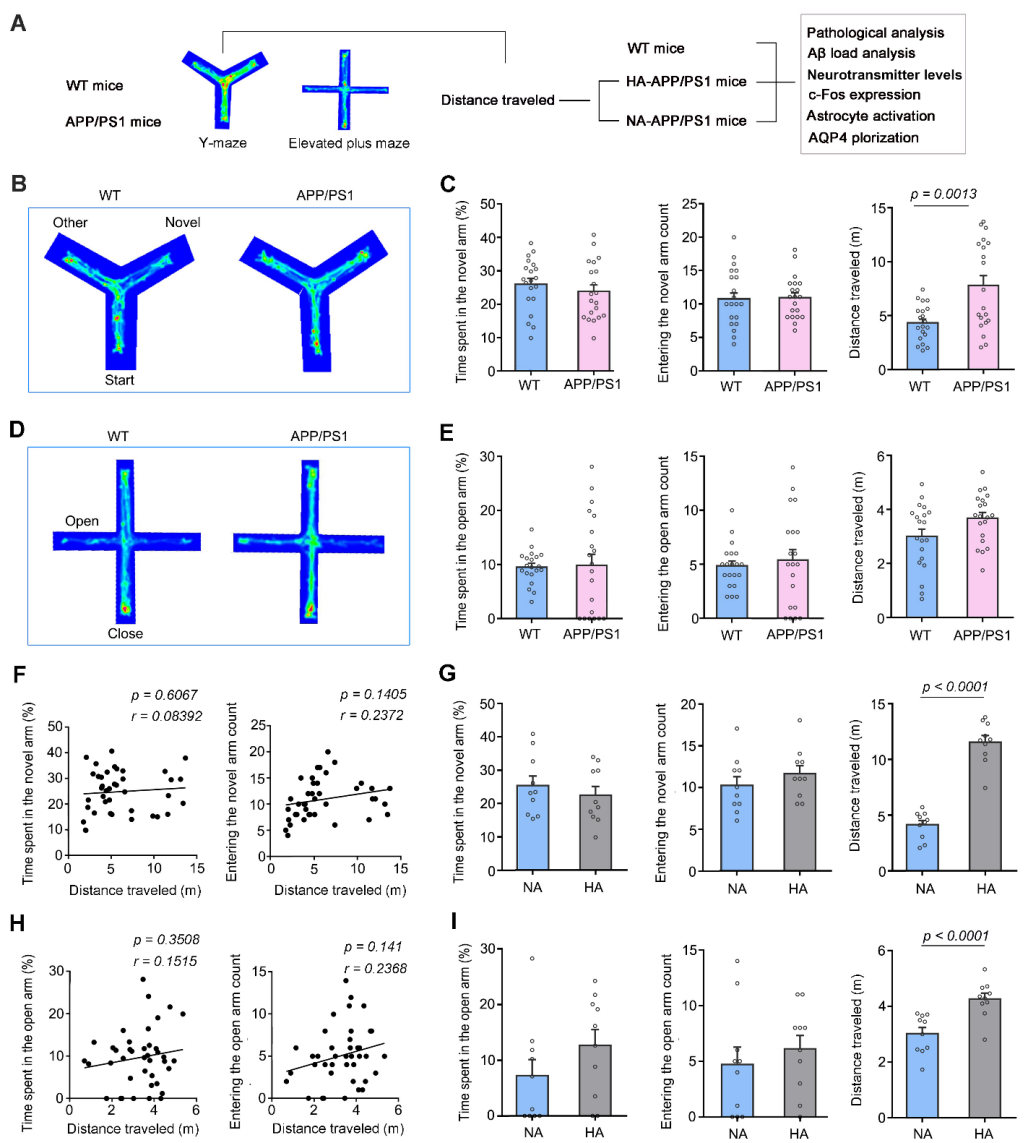
**A proportion of 4-month-old APP/PS1 mice was hyperactive during Y-maze and elevated plus maze tests**. (**A**) Schematic diagram of the experimental procedure showing that WT mice and APP/PS1 mice received behavior tests followed by pathological analyses. (**B**) Representative tracing of mouse movement during 5-min Y-maze test. (**C**) Percentage of time spent in the novel arm (left panel), number of entries into the novel arm (middle panel), and total distance traveled (right panel) during the Y-maze test. (**D**) Representative tracing of mouse movement during 5-min elevated plus maze (EPM) test. (**E**) Percentage of time spent in the open arm (left panel), number of entries into the open arm (middle panel), and total distance traveled (right panel) during the EPM test. (**F**) Correlation analysis of total distance traveled during the Y-maze test and percentage of time spent in the novel arm (left panel), and number of entries into the novel arm (right panel), respectively. (**G**) Y-maze test showing that time spent (left panel) and number of entrance to the novel arm (middle panel) were comparable between subgroups of APP/PS1 mice with normal-activity (NA) and high-activity (HA) (right panel). (**H**) Correlation analysis of total distance traveled during the EMP test, percentage of time spent in the open arm (left panel) and number of entries into the open arm (right panel). (**I**) In the EPM test, HA-APP/PS1 mice exhibited similar dwelling time (right panel) and entry number (middle panel), but a longer distance traveled (right panel), compared with NA-APP/PS1 mice. Data represent mean ± SEM. Data in (**C, E**) from 20 mice per group and (**G, I**) from 10 mice per group were analyzed by Student’s t-test. Data in (**F, H**) from 40 mice were analyzed by Pearson correlation analysis.

**Figure 2.**
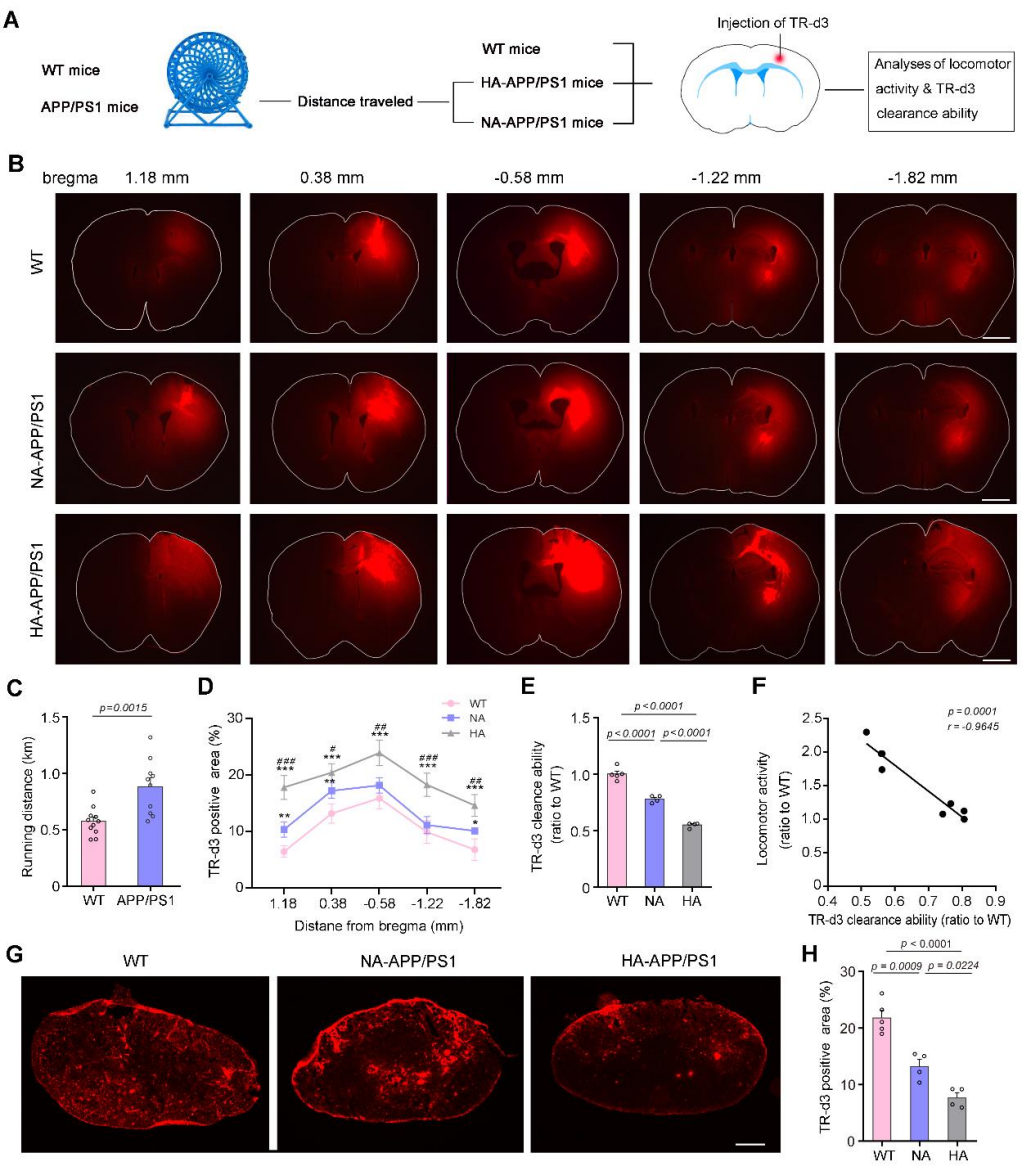
**Hyperactivity associated with low glymphatic transport ability in APP/PS1 mice**. (**A**) Schematic diagram of the experimental procedure showing mice received spontaneous locomotor testing followed by glymphatic transport analysis. (**B**) Representative images of a series of coronal sections show that TR-d3 clearance was markedly impaired in HA-APP/PS1 brains compared to NA-APP/PS1 brains. Scale bar, 500 μm. (**C**) Movement distance of APP/PS1 mice running in wheel cages for 2 hours was significantly higher than that of WT mice. (**D**) Forty-five minutes after injection into the cerebral cortex, TR-d3 clearance was evaluated by quantification of the percentage of its positive area in coronal sections with different distances from the bregma. (**E**) TR-d3clearance capability defined as 100% minus the TR-d3 positive area percentage and shown as a ratio to the WT level, was significantly lower in hyperactivity (HA)-APP/PS1 mice than that in normal activity (NA)-APP/PS1 mice and WT mice. (**F**) Relative locomotor activity (the ratio of running distance between APP/PS1 mice and WT mice) was negatively correlated to TR-d3 clearance ability. (**G**) Representative images showing distribution of TR-d3 within the dcLNs. Scale bar, 100 μm. (**H**) Quantification of the area percentage of TR-d3 fluorescence in the dcLNs after intracortical injection. Data represent mean ± SEM. Data in (C) from 11 WT mice and 10 APP/PS1 mice were analyzed by Student’s t-test. Data in (D) from 5 WT mice, 4 NA-APP/PS1 mice and 4 HA-APP/PS1 mice were analyzed by repeated measures one-way ANOVA with Tukey’s post hoc test. Data in (E and H) from five sections per mouse with 5 WT mice, 4 NA-APP/PS1 mice and 4 HA-APP/PS1 mice were analyzed by one-way ANOVA with Tukey’s post hoc test. Data in (F) from 8 mice were analyzed by Pearson correlation analysis.

AQP4 knockout (AQP4*^-/-^*) APP695/PS1-DE9 (APP/PS1) transgenic mice were generated as previously described [23]. Both male and female AQP4*^-/-^*/APP/PS1 mice, APP/PS1 mice, and their wild-type (WT) controls with C57BL/6 genetic background, were bred and raised to 4 months old in a pathogen-free facility under a 12 h:12 h light: dark cycle environment and fed with regular rodent’s chow and sterilized tap water ad libitum. The first subgroup of APP/PS1 mice (10 males and 10 females) and WT mice (10 males and 10 females) received the Y maze and elevated plus maze (EPM) tests and AD-related pathological analysis (Fig. 1A); the second subgroup of APP/PS1 mice (6 males and 5 females) and WT mice (5 males and 5 females) were used for motor ability measurement, followed by evaluation of glymphatic transport function (Fig. 2A); and the third subgroup of APP/PS1 mice (5 males and 5 females) and WT mice (6 males and 8 females) were given the open field test to compare their exploratory behavior and general activity. APP/PS1 mice (5 males and 5 females) and AQP4*^-/-^*/APP/PS1 mice (8 males and 7 females) were also used to compare their memory function, anxiety level, brain Aβ load and glutamatergic neuron activation. All animal experiments were conducted in accordance with the regulations of the Animal Ethics Committee of Nanjing Medical University and the guidance requirements of international animal welfare standards.

### Y-maze test

The Y-maze test was performed to evaluate mouse short-term spatial memory [23, 25]. The Y-maze (length: 27 cm; width: 9 cm; height: 24 cm) consists of three identical arms with different designs as visual markers for mice. The experimental process contains a 5 min-training stage and a 5 min-testing stage. During the first stage, the mouse was only allowed to move in the start arm and another arm, with the novel arm (NA) blocked by a baffle. Two hours later, the NA was opened, and the mouse was allowed to explore all three arms. The percentage of time spent in the NA, the number of entries into the NA and the total distance traveled during the test were calculated by Topscan Software (TopScan, CleverSys, Inc., Reston, USA).

### Elevated plus maze test

On the next day after Y-maze test, the EPM test was performed to evaluate anxiety-like behavior [26]. It consists of a cross-shaped maze including two open arms (length: 35 cm; width: 5 cm) and two closed arms (length: 35 cm; width: 5 cm; height: 15 cm) that are elevated 50 cm above the ground. Each mouse was gently placed in the center of the cross area, and the percentage of time spent in the open arm, the number of open arm entries and the total distance traveled during 5-min testing period were analyzed by Topscan Software.

### Locomotor activity test

To conduct the locomotor activity test, a mouse was placed into a 12.5 cm-diameter running wheel (Ji Biao Aquarium Co., Ltd., Jinhua, China) equipped with a modified pedometer (Pu Ning Electric Co., Ltd., Leqing, China). Rotation number was recorded for 2 hours, and running distance was calculated.

### The open field test

The open field consisted of a square black Plexiglas box (length: 60 cm; width: 60 cm; height: 25 cm), with an outlined center area (30 cm × 30 cm). Each mouse was gently placed in the middle of box and allowed to move freely within the box for 5 min [26]. The time spent in the center area, times of entering the center area and total distance traveled during the test were measured.

The aforementioned behavioral tests were performed during the illuminated part of the diurnal cycle (08:00 AM-11:00 AM), under conditions of dim light (~10 lx) and low noise. Mouse activity in the Y-maze, EPM and open field was collected by a digital video camera connected to a computer-controlled system (Beijing Sunny Instruments Co. Ltd, China). All tests were performed by two investigators who were blind to the treatment schedule.

### Injection of fluorescent tracer

The day after behavioral testing, WT and APP/PS1 subgroup mice were anesthetized by 1% pentobarbital sodium (40 mg/kg body weight) and positioned on a stereotaxic apparatus. Following exposure of the skull, a burr hole was made with a hand-held drill on coordinates anterior/posterior: +0.5 mm, dorsal/ventral: +1.8 mm, medial/lateral: +2.0 mm from bregma. A one-microliter volume of 0.5% Texas Red-dextran-3 (TR-d3, molecular weight: 3 kDa, Invitrogen, Carlsbad, USA, Cat# D3328) was slowly injected through a glass micropipette attached to a Hamilton micro-syringe at a rate of 0.1 μL/min using a syringe driver [24]. The micropipette remained in place for 5 min, and then was withdrawn very slowly to avoid any possible backflow. Forty-five min later, mice received a second supplementary anesthesia. Brains and deep cervical lymph nodes (dcLNs) were quickly removed and fixed overnight in 4% paraformaldehyde at 4°C.

### Sample preparation

Following behavioral testing, mice were intraperitoneally injected with 1% pentobarbital sodium and then transcardially perfused with 0.9% saline solution, followed by 4% paraformaldehyde for 5 min. Brain tissues were harvested, post-fixed overnight, and then serially dehydrated in graded ethanol solutions and embedded in paraffin. Brain sagittal sections were sliced into 5-µm-thickness using a sliding microtome (SM2000R; Leica, Solms, Germany). For fluorescent tracer experiment, the forebrain was sliced into a series of coronal sections at 100 µm using a vibratome (VT1200; Leica, Solms, Germany). Sections from 2.5 mm anterior to 2.5 mm posterior to bregma were sequentially slide-mounted. The dcLNs were cut at 30 μm on a cryostat (Leica) and mounted onto gelatin-coated slides. For immunoblotting, enzyme-linked immunosorbent assay (ELISA) and high-performance liquid chromatography (HPLC) analysis, anesthetized mice were sacrificed by cervical dislocation. Brains were quickly dissected, frozen in liquid nitrogen, and then stored at -80 °C awaiting analysis.

### HPLC analysis

HPLC analysis was performed to measure concentrations of amino acid neurotransmitters in the mouse frontal cortex, including aspartate (Asp), glutamate (Glu), glutamine (Gln), glycine (Gly), taurine (Tau), serine (Ser) and gamma-amino butyric acid (GABA) [27]. Cortical tissues were homogenized and centrifuged at 4°C and 20000 *g* for 30 min to collect supernatant. Derivatization of amino acids was separated using a reverse phase C18 column (150 mm × 4.6 mm, 5 µm; Waters Corp., Milford, USA), a HPLC RF-10A fluorescence detector (Shimadzu Corp., Kyoto, Japan), and a liquid chromatography LC-10AD (Shimadzu Corp., Kyoto, Japan). The emission wavelength was set at 425 nm and the excitation wavelength at 328 nm. The flow rate was set at 1.0 mL/min. An external calibration curve was used to express the final amount in the tissue sample as microgram per gram (μg/g) wet tissue for amino acids. Mean value of each sample was obtained from triplicate independent experiments.

### Immunofluorescence staining

Sections were dewaxed with xylene and ethanol, washed with distilled water, and incubated in citrate buffer solution (pH 6.0) for antigen retrieval. Slices were incubated overnight with a mixture of mouse monoclonal anti-glial fibrillary acidic protein (GFAP) antibody (1:1000; Millipore, Burlington, USA; Cat# MAB360) plus rabbit polyclonal anti-AQP4 antibody (1:400; Millipore; Cat# AB3594), mouse monoclonal anti-Aβ_1-16_ (1:1000; Biolegend, San Diego, USA; Cat# 803001) plus rabbit polyclonal anti-NeuN (1:400; Millipore; Cat# MABN140) or mouse monoclonal anti-c-Fos antibody (1:400; Santa Cruz Biotechnology, USA; Cat# SC-166940) plus goat polyclonal anti-vesicular glutamate transporter 1 (VGLUT1) antibody (1:400; Synaptic Systems, Goettingen, Germany; Cat# 135307) at 4°C overnight. After rinsing, the sections were incubated with a mixture of Alexa Flour 555 donkey anti-mouse IgG (1:1000; Thermo Fisher Scientific (China) Co., Ltd., Shanghai, China; Cat# A31570), and Alexa Flour 488 donkey anti-rabbit IgG (1:1000; Thermo Fisher; Cat# A21206) or Alexa Flour 555 donkey anti-goat IgG (1:1000; Thermo Fisher; Cat# A21432) and Alexa Flour 488 donkey anti-mouse IgG (1:1000; Thermo Fisher; Cat# A21202). Finally, sections were rinsed, then incubated for 5 min in 4’,6-diamidino-2-phenylindole (DAPI) (1:1000; Thermo Fisher; Cat# D1306) and coverslipped with anti-fluorescent quencher. Excitation and emission wavelength was set to 555 and 565 nm for Alexa Flour 555, 495 and 519 nm for Alexa Flour 488, and 358 and 461 nm for DAPI, respectively. Immunofluorescence controls were taken on some mouse brain sections. Primary antibodies were omitted or replaced with an equivalent concentration of normal mouse, rabbit or goat serum. All sections were immuno-negative.

### Thioflavine-S staining

Deparaffinized sections were incubated with 1% thioflavine-S (Millipore Sigma, St. Louis, USA; Cat# 1326-12-1) for 5 min [25]. 70% ethanol was then used for 5 min to differentiate, followed by rinsing with distilled water. Brain sections were coverslipped with anti-fluorescent quencher.

### Image processing

Sections were visualized using a Leica digital microscope or a Zeiss LSM710 confocal microscope, captured with constant exposure time, offset, and gain for each staining marker. ImageJ (NIH) was used to analyze the area percentage of positive signals of TR-d3 in the dcLNs and the whole coronal sections at +1.18, 0.38, -0.58, -1.22 and -1.82 mm from anterior to posterior, relative to the bregma. The area percentage of Thioflavine-S, Aβ_1-16_ and GFAP fluorescent signals in the frontal cortex was also measured. Minimum and maximum intensity were set to be constant using interest gray threshold analysis. The number of vGLUT-1/c-Fos double-positive cells and GFAP positive cells were counted in four random images at 400× magnification (0.44 × 0.44 mm^2^) of the motor cortex. To analyze the expression and polarization of AQP4, images of the motor cortex were captured. The immunofluorescence intensity of AQP4 in the cortex extending 500 µm below the brain surface was detected. The polarity of AQP4 was calculated by comparing the expression intensity of AQP4 at the pial surface and perivascular domains versus adjacent parenchymal regions [25]. Three brain sections in each set were averaged for each mouse, and 4-6 mice were averaged for each group. All quantification was done blind to animal genotype.

### ELISA

Frontal cortex samples were homogenized and sonicated in ice-cold TBS buffer containing 0.5 mM PMSF, 0.5 mM benzamidine, 1.0 mM DTT and 1.0 mM EDTA, then centrifuged at 100,000 *g* for 1 hour [28]. Supernatants were set aside for measurements of soluble Aβ_1-40_ (R&D Systems; Minneapolis, USA; Cat# DAB140B) and Aβ_1-42_ (R&D Systems; Cat# DAB142). The above indexes were quantified with ELISA kits according to the manufacturer’s instructions. Mean value of each sample was obtained from triplicate independent experiments.

### Western blotting

Homogenized cortical tissues were lysed in RIPA buffer (containing 1 mM PMSF, 1× Halt Protease and Phosphatase Inhibitor Cocktail (Thermo)) and centrifuged at 13,000 *g* for 15 min at 4°C. The extracts were boiled at 95°C for 5 min with SDS loading buffer. The samples were resolved on 10%-15% SDS-PAGE gels, and then transferred onto polyvinylidene fluoride membranes. After blocking in 5% defatted milk for 1 hour, the membrane was incubated at 4°C overnight with appropriate dilutions of following primary antibodies: rabbit anti-AQP4 antibody (1:400; Millipore; Cat# AB3594), mouse GFAP antibody (1:1000; Millipore, Burlington, USA; Cat# MAB360), rabbit anti-a disintegrin and metalloproteinase 10 (ADAM10) antibody (1:1000; Millipore; Cat# AB19026), mouse anti-β-site amyloid precursor protein-cleaving enzyme 1 (BACE1) antibody (1:1000; Millipore, #MAB5308), rabbit anti-PS1 antibody (1:1000; Sigma-Aldrich, #PRS4203), rabbit anti-neprilysin (NEP) antibody (1:1000; Millipore, #AB5458), rabbit anti-insulin-degrading enzyme (IDE) antibody (1:1000; Abcam, #ab32216), rabbit anti-APP (C-terminal) antibody (1:1000; Sigma-Aldrich, #A8717) or mouse anti-glyceraldehyde-3-phosphate dehydrogenase (GAPDH) antibody (1:3000; Proteintech; #6004-1-Ig). Following TBST washing, the membrane was incubated with goat anti-mouse IgG conjugated with horseradish peroxidase (HRP; 1:2000; ZSGB-BIO, #AB2305) or goat anti-rabbit IgG conjugated with HRP (1:2000; ZSGB-BIO, #AB2301) for 1 hour at room temperature, then imaged with the GE imaging system (Image Quant LAS 4000 mini, version 1.2). The integrated optical density (IOD) of each protein band was normalized with the respective GAPDH band and the average value was obtained from duplicate independent experiments per mouse. Final results are presented as a relative expression ratio to the WT level.

### Statistical analysis

All data involved in this experiment are represented by mean± SEM. Graphpad Prism 6.0 was used for data analysis. Sample size estimation was based on a priori power analysis and previously reported data [18, 24, 26]. If statistical data did not obey normal distribution and the homogeneity of variance, a non-parametric test (Kruskal-Wallis test) was used. The Student’s t-test was used comparing WT mice and APP/PS1 mice, APP/PS1 mice with normal activity versus hyperactive APP/PS1 mice, as well as APP/PS1 mice versus AQP4*^-/-^*/APP/PS1 mice. One-way ANOVA with Tukey’s post hoc test (or Kruskal-Wallis test) were used to compare the data among WT mice, hyperactive APP/PS1 mice and normally activate APP/PS1 mice. The percentage of TR-d3 positive area in coronal sections with different distances from the bregma was compared among the above three animal groups by using the repeated measure ANOVA with Tukey’s post hoc test. Pearson correlation analysis was used to examine the correlation between distance traveled and memory, anxiety or TR-d3 clearance ability related indexes. All statistical analyses were indicated in the figure legends. *P* < 0.05 indicated that the difference was statistically significant.

## RESULTS

### Hyperactivity of 4-month-old APP/PS1 mice

APP/PS1 mice, the most common AD animal model, not only exhibit an age-dependent decline in cognitive ability, but also simulate BPSD-like behaviors of AD patients [29-35]. However, whether BPSD-like abnormalities occur in the early stage of this AD mouse model remains unclear. Therefore, we examined cognitive function and anxiety-like behavior of APP/PS1 mice at 4 months old by the Y-maze and EPM testing, respectively. In the Y-maze, APP/PS1 mice displayed normal short-term memory, since the number of entrances and time spent in the novel arm were similar to those in WT mice (*p* = 0.9671, *p* = 0.4021, respectively, n = 20 per group, Student’s t-test). However, APP/PS1 mice exhibited an increased total distance traveled during the testing period (*p* = 0.0013, n = 20 per group, Student’s t-test; Fig. 1B, C). Similarly, APP/PS1 mice did not show anxiety-like performance in the EMP, as revealed by no changes in the number of entrances and residence time in the open arm, compared with WT mice (*p* = 0.5821, *p* = 0.8738, respectively, n = 20 per group, Student’s t-test). However, they traveled a longer distance than WT mice (*p* = 0.0421, n = 20 per group, Student’s t-test; Fig. 1D, E). The movement distance of WT mice and APP/PS1 mice in the Y-maze was not correlated with the time spent in the new arm or number of entrances into the new arm (*p* = 0.6067, *r* = 0.08392; *p* = 0.1405, *r* = 0.2372, respectively, n = 40, Pearson correlation analysis; Fig. 1F). Similarly, movement distance of WT mice and APP/PS1 mice in the EPM also did not correlate with the open arm residence time or entry times (*p* = 0.3508, *r* = 0.1515; *p* = 0.141, *r* = 0.2368, respectively, n = 40, Pearson correlation analysis; Fig. 1H).

Moreover, movement distance displayed obvious inter-individual variability among APP/PS1 mice. Therefore, they were divided into a hyperactivity group (HA-APP/PS1) and normal activity group (NA-APP/PS1) according to the total movement distance in the Y-maze. The average distance of HA-APP/PS1 mice was 50% higher than that of WT mice (*p* < 0.0001, WT, n = 20, HA-APP/PS1, n =10, Student’s t-test; Fig. 1C, G). These HA-APP/PS1 mice also showed an increase of distance traveled in the EPM (*p* < 0.0001, *vs.* NA-APP/PS1, n = 10 per group, Student’s t-test; Fig 1I). However, the above short-term memory and anxiety-like indexes were not significantly different between HA-APP/PS1 mice and NA-APP/PS1 mice (Y maze: time spent: *p* = 0.4635; entering number: *p* = 0.3405; EPM: time spent: *p* = 0.2009; entering number: *p* = 0.4899, n = 10 per group, Student’s t-test; Fig. 1G, I). There was also no correlation between total distance travelled and cognitive or anxiety-like behavior measures in hyperactive APP/PS1 mice (Y-maze: distance & time spent: *p* = 0.5093, *r* = 0.2372; distance & entering number: *p* = 0.7652, *r* = 0.1086; EPM: distance & time spent: *p* = 0.5750, *r* = 0.2024; distance & entering number: *p* = 0.7881, *r* = 0.09781, n = 10, Pearson correlation analysis; Supplementary Fig. 1A, B). We further confirmed that some APP/PS1 mice were hyperactive in the wheel experiment during the two-hour testing, with the running distance of these mice being approximately 1.5-fold higher than that of WT mice (*p* = 0.0015, WT, n = 11; APP/PS1, n = 10, Student’s t-test; Fig. 2A, C).

We also performed the open field test to compare exploratory behavior and general activity between APP/PS1 mice and WT controls. Percentage of time spent in the central area, number of entries into the central area, and total distance traveled were comparable between the two genotypes (*p* = 0.6359, *p* = 0.5475, *p* = 0.2282, respectively, WT, n = 10, APP/PS1, n =14, Student’s t-test). However, there was more obvious inter-individual variability of movement distance in APP/PS1 mice, as compared with WT mice (Supplementary Fig. 2A, B). Therefore, these APP/PS1 mice were further divided into HA group and NA group (*p* = 0.0019, NA, n = 8, HA, n= 6, Student’s t-test). The percentage of time spent in the central area and number of entries into the central area were not different between HA-APP/PS1 mice and NA-APP/PS1 mice (*p* = 0.4545, *p* = 0.1734, respectively, NA, n = 8, HA = 6 Student’s t-test; Supplementary Fig. 2C). Together, these results suggest that hyperactivity occurs in a subset of 4-month-old APP/PS1 mice, which is not related to either cognitive performance or anxiety-like extent.

**Figure 3.**
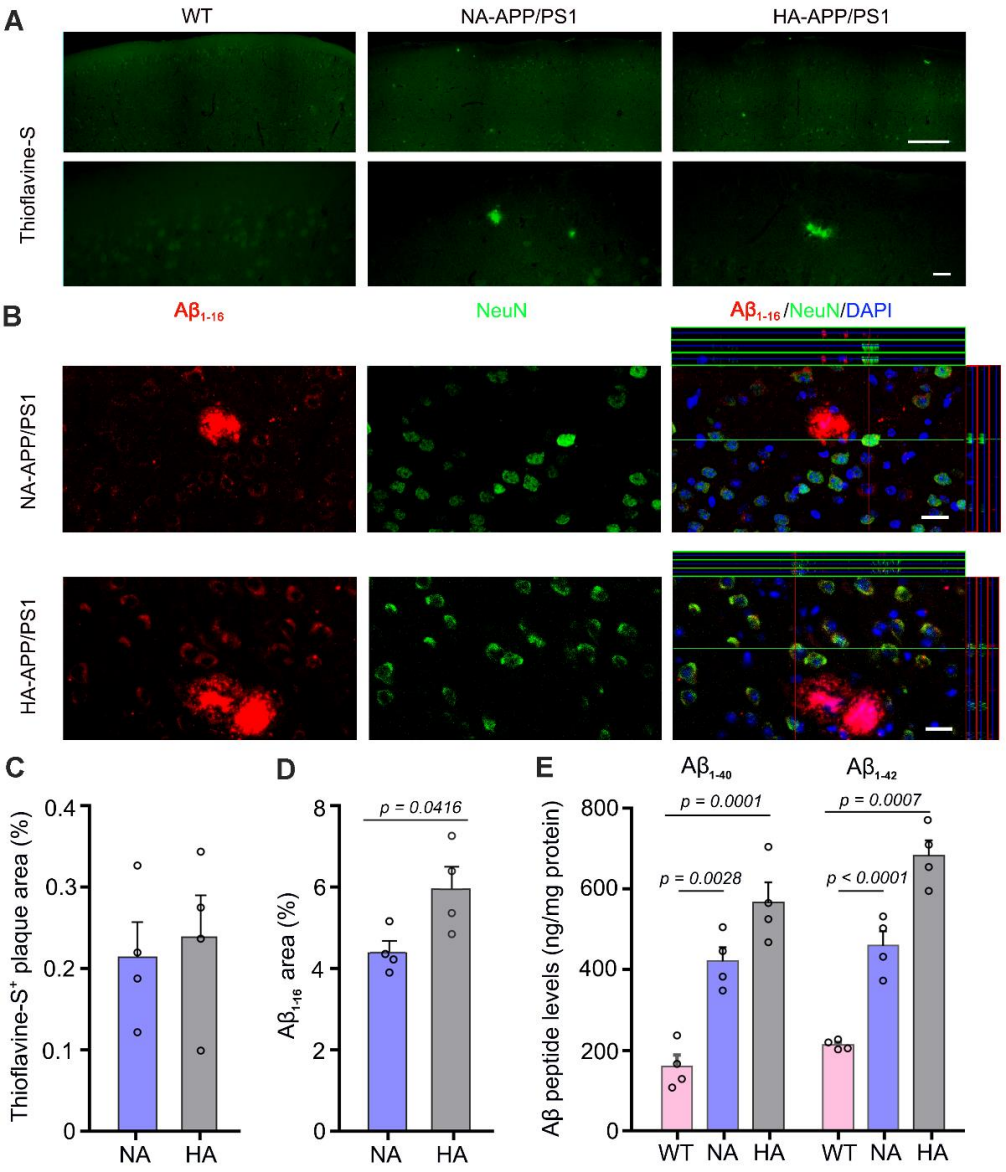
**Analysis of Aβ load in APP/PS1 mice with hyperactivity and normal activity**. (**A**) Representative images display Thioflavine-S positive plaques scattered in the frontal cortex of APP/PS1 mice with hyperactivity (HA) and normal activity (NA). Scale bar, 200 μm (upper panel); 20 μm (lower panel). (**B**) Representative images of Aβ_1-16_ immunofluorescence revealed that Aβ plaques were observed in both HA-APP/PS1 mice and NA-APP/PS1 mice, but intraneuronal accumulation of Aβ was more obvious in HA-APP/PS1 mice. Scale bar, 15 μm. (**C**) The percentage of Thioflavine-S positive plaque areas of WT mice and APP/PS1 mice. (**D**) HA-APP/PS1 mice exhibited a higher percentage of Aβ_1-16_ positive area than NA-APP/PS1 mice. (**E**) ELISA analysis showed that the concentrations of Aβ_1-40_ and Aβ_1-42_ were higher in HA-APP/PS1 mice, compared with NA-APP/PS1 mice and WT mice. Data represent mean ± SEM from 3 sections per mouse (in C, D) or triplicate independent experiments (in E) with four mice per group. Data in (C, D) were analyzed by Student’s t-test. Data in (E) were analyzed by one-way ANOVA with Tukey’s post hoc test.

**Figure 4.**
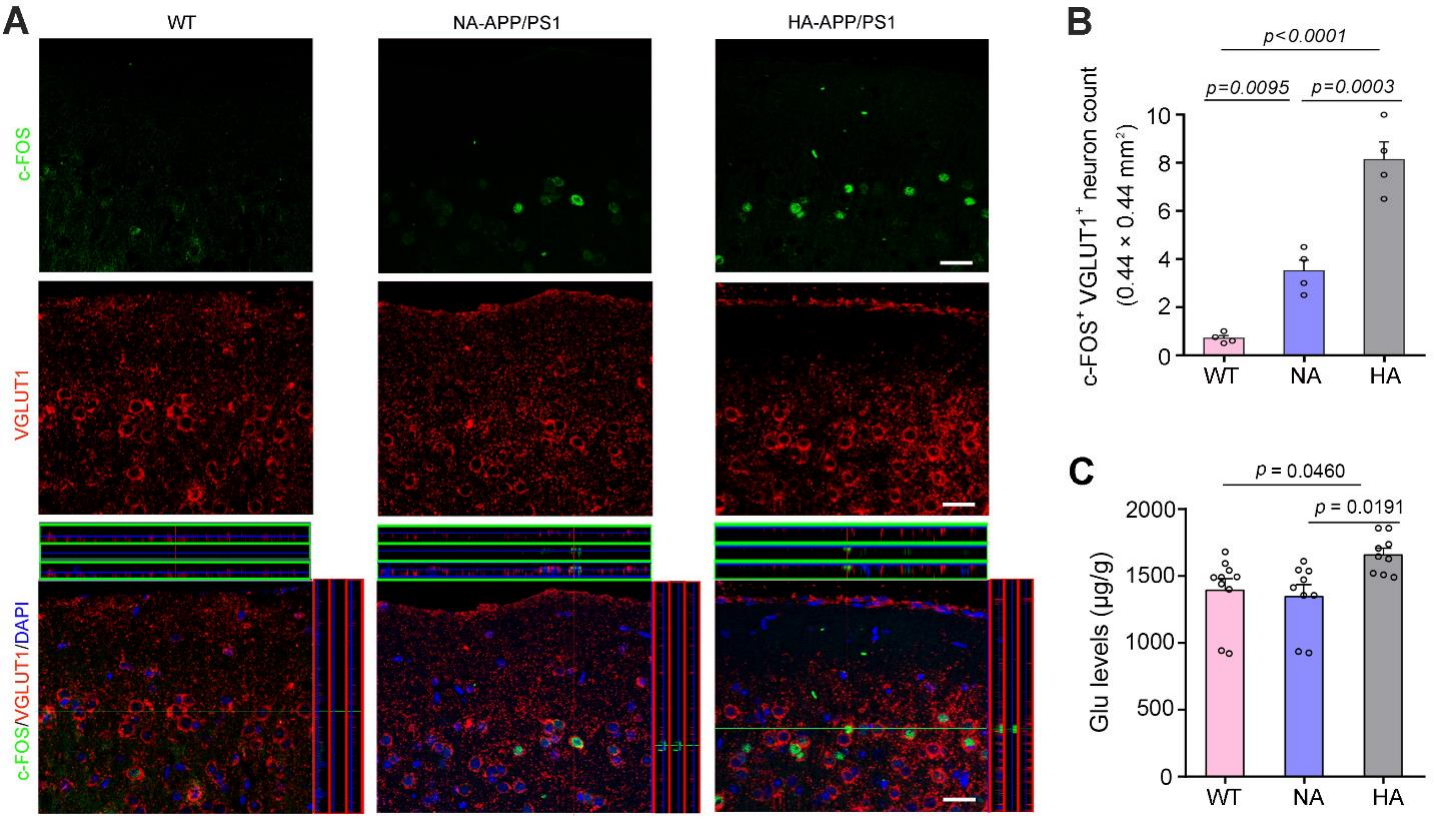
**Analysis of glutamatergic neuron activation in the motor cortex of APP/PS1 mice with hyperactivity and normal activity**. (**A**) Representative images showed that c-Fos, a marker of neuronal activity, was expressed by a small proportion of VGLUT1 positive neurons in the motor cortex of APP/PS1 mice with hyperactivity (HA) and normal activity (NA). Scale bar, 15 μm. (**B**) Quantification of c-Fos and VGLUT1 double labeled neurons per high magnification field (0.44 mm × 0.44 mm). (**C**) Quantification of Glu concentrations measured by HPLC. Data represent mean ± SEM of 3 sections per mouse with 4 mice per group (B) or triplicate independent experiments with 10 WT mice, 9 NA-APP/PS1 mice and 9 HA-APP/PS1 mice (C). Data were analyzed by one-way ANOVA with Tukey’s post hoc test.

### Increases in intraneuronal Aβ load and neuronal activation of APP/PS1 mice with hyperactivity

We then investigated the pathological mechanisms of hyperactivity in APP/PS1 mice. Thioflavine-S staining showed sporadic deposition of Thioflavine-S positive fibrillary plaques in the cerebral cortex 4-month-old APP/PS1 mice (Fig. 3A), consistent with previous reports [36-38]. However, there was no significant difference in the area percentage of positive plaques between NA-APP/PS1 mice and HA-APP/PS1 mice (*p* = 0.7272, n = 4 per group, Student’s t-test; Fig. 3C). However, HA-APP/PS1 mice had increased intraneuronal Aβ load in the motor cortex, as revealed by immunostaining for Aβ_1-16_ (*p* = 0.0416, n = 4 per group, Student’s t-test; Fig. 3B, D). APP/PS1 mice with high activity in the open field test also showed high amyloid accumulation (*p* = 0.0394, *vs.* NA-APP/PS1, n = 4 per group; Student’s t-test; Supplementary Fig. 2D, E). ELISA analysis further confirmed that Aβ_1-40_ and Aβ_1-42_ levels in the frontal cortex were higher in HA-APP/PS1 mice than NA-APP/PS1 mice (F_2, 9_ = 27.81, *p* = 0.0001, HA *vs.* NA, *p* = 0.0460; F_2, 9_ = 60.48, *p* < 0.0001, *p* = 0.0014, respectively, n = 4 per group, one-way ANOVA with Tukey’s post hoc test; Fig. 3E).

It is known that oligomeric Aβ may induce neuronal hyper-excitability even in the early phases of AD patients [39-43] and mouse models of AD [44-47]. Therefore, we observed c-Fos expression, a marker for neuronal excitation, in glutamatergic neurons of the frontal cortex. As expected, the number of c-Fos and VGLUT1 double labeled neurons in HA-APP/PS1 mice was significantly higher than that in NA-APP/PS1 mice (F_2, 9_ = 35.89, *p* < 0.0001, HA *vs.* NA, *p* = 0.0003, n = 4 per group, one-way ANOVA with Tukey’s post hoc test; Fig. 4A, B). In addition, we found HA-APP/PS1 mice had higher levels of Glu in the frontal cortex than NA-APP/PS1 mice (F_2, 25_ = 4.998, *p* = 0.0149, HA *vs.* NA, *p* = 0.0191, WT, n = 10; NA, n = 9; HA, n = 9, one-way ANOVA with Tukey’s post hoc test; Fig. 4C), while other amino acids such as GABA, Tau, Asp, Ser, Gln and Gly were no different from each other (F_2, 25_ = 0.7591, *p* = 0.4786; F_2, 25_ = 4.911, *p* = 0.0159; F_2, 25_ = 3.580, *p* = 0.0429; F_2, 25_ = 0.8468, *p* = 0.4407; F_2, 25_ = 3.207, *p* = 0.0576; F_2, 25_ = 1.302, *p* = 0.2898, respectively, WT, n = 10; NA, n = 9; HA, n = 9; one-way ANOVA with Tukey’s post hoc test; Supplementary Fig. 3). Together, the above results suggest that the hyperactivity of APP/PS1 mice may be related to increased excitability of glutamatergic neurons caused by intraneuronal Aβ accumulation.

**Figure 5.**
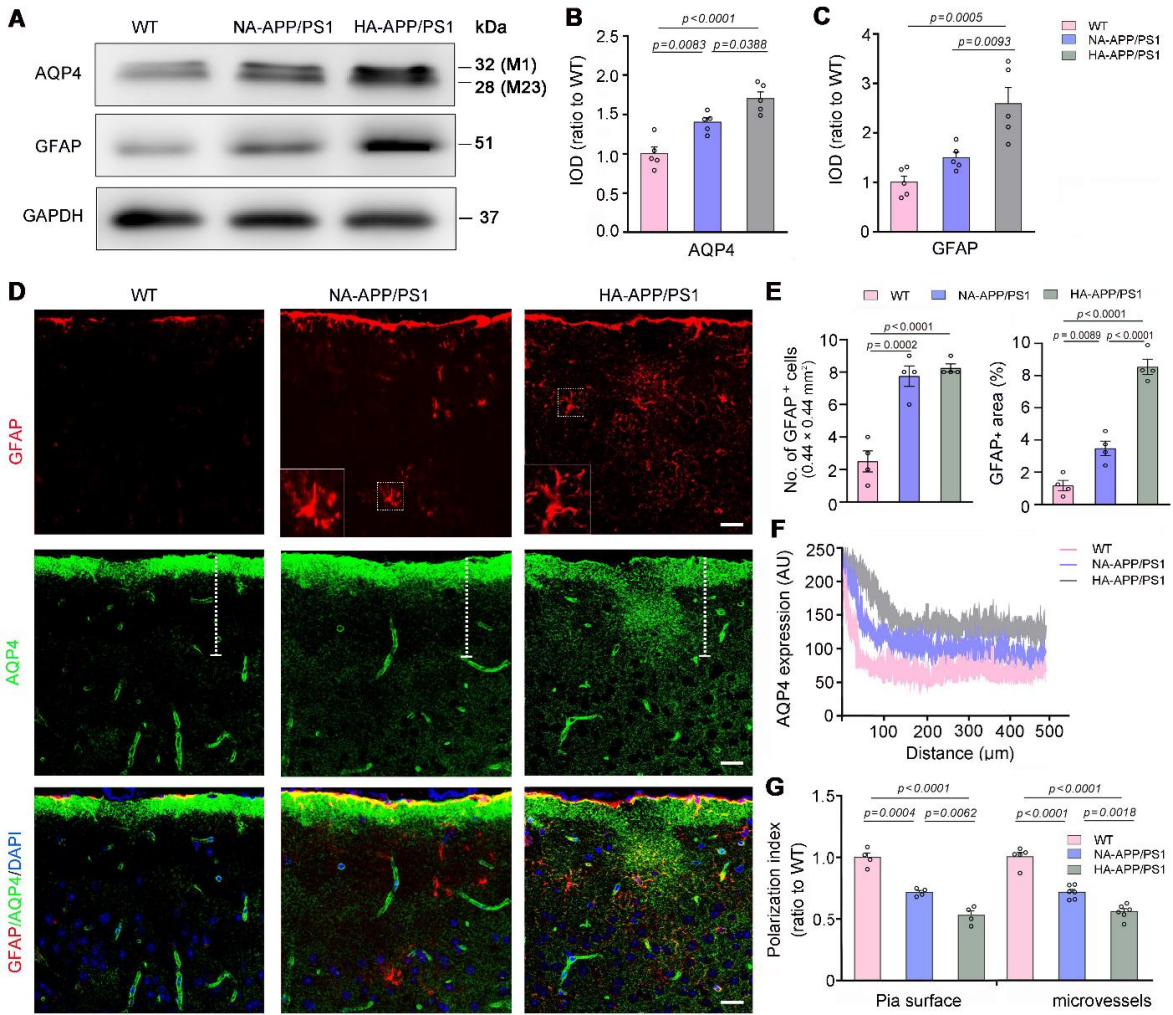
**Analysis of astrocyte activation and AQP4 expression pattern in the motor cortex of APP/PS1 mice with hyperactivity and normal activity**. (A-C) Representative Western blot and densitometric analysis of AQP4 and GFAP in the motor cortex of WT mice and APP/PS1 mice with hyperactivity (HA) and normal activity (NA). The integrated optical density (IOD) was shown as a relative expression ratio to the WT level. (**D**) Representative images of GFAP and AQP4 double immunofluorescence in the motor cortex of WT mice, HA-APP/PS1 mice and NA-APP/PS1 mice. Activated astrocytes with hypertrophic cell bodies and intensely stained processes (outlined area) were observed in APP/PS1 mice. Scale bar, 15 μm. (**E**) Quantification of the number of GFAP positive cells (left panel) and the percentage of GFAP positive area (right panel) in the motor cortex. (**F**) Quantitative analyses of immunofluorescence intensity of AQP4 in the frontal cortex of the above groups. (**G**) Quantitative analyses of AQP4 polarization abutting pia maters and microvessels. Data represent mean ± SEM. Data in (B and C) were from duplicate independent experiments of 5 mice per group, in (E) from 3 sections per mouse, 4 mice per group, and in (G) from 3 sections per mouse with 4 mice per group for pia surface analysis, and 6-8 microvessels per mouse with 5 WT mice, 6 NA-APP/PS1 mice and 6 HA-APP/PS1 mice for microvessels analysis. The number of GFAP positive cells (the left panel in E) was analyzed by the one-way ANOVA with Kruskal-Wallis test. The other data were analyzed by the one-way ANOVA with Tukey’s post hoc test.

### Hyperactivity was associated with AQP4 mislocalization in APP/PS1 mice

Accumulation of Aβ within the brain is due to an imbalance between its generation and clearance [48, 49]. Therefore, we examined the expression levels of Aβ metabolism and clearance related proteins in the frontal cortex by Western blotting. HA-APP/PS1 mice and NA-APP/PS1 mice had high levels of APP and PS1 compared to WT mice (APP: HA *vs.* WT, *p* = 0.0285; NA *vs.* WT, *p* = 0.0482; PS1: HA *vs.* WT, *p* = 0.0372; NA *vs.* WT, *p* = 0.0372, respectively; n = 4 per group, Kruskal-Wallis test; Supplementary Fig. 4A, B). The levels of ADAM10 and BACE1 were comparable among the three groups (F_2, 9_ = 3.163, *p* = 0.0911; F_2, 9_ = 2.33, *p* = 0.1529, respectively; n = 4 per group, one-way ANOVA with Tukey’s post hoc test; Supplementary Fig. 4A, B). Furthermore, the expression levels of Aβ-related degradation enzymes NEP and IDE were similar among the three groups (F_2, 9_ = 2.1, *p* = 0.1785; F_2, 9_ = 0.5843, *p* = 0.5773, respectively; n = 4 per group, one-way ANOVA with Tukey’s post hoc test; Supplementary Fig. 4A, B).

**Figure 6.**
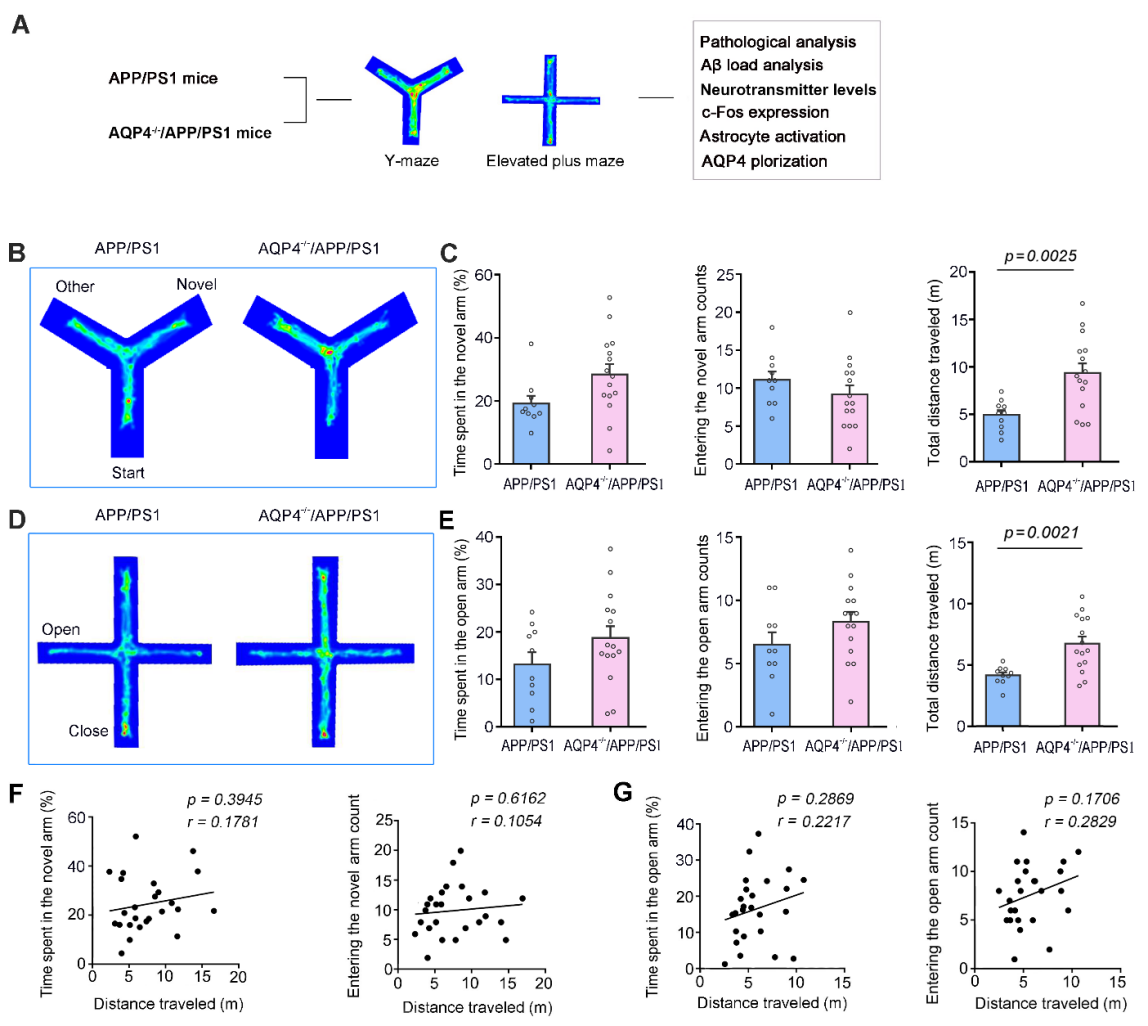
**AQP4 deletion resulted in hyperactivity of 4-month-old APP/PS1 mice during Y-maze and elevated plus maze tests**. (**A**) Diagram showing APP/PS1 mice and AQP4*^-/-^*/APP/PS1 mice that received behavior tests followed by pathological analyses. (**B**) Tracing of mouse movement during the Y-maze test. (**C**) Percentage of time spent in the novel arm (left panel), number of entries into the novel arm (middle panel) and travelling distance (right panel) during the Y-maze test. (**D**) Mouse movement tracing during the elevated plus maze (EPM) test. (**E**) Percentage of time spent in the open arm (left panel), number of entries into the open arm (middle panel) and travelling distance (right panel) during the EPM test. (**F**) Correlation analysis of total distance traveled during the Y-maze test and percentage of time spent in the novel arm (left panel), and number of entries into the novel arm (right panel). (**G**) Correlation analysis of total distance traveled during the EPM test and percentage of time spent in the open arm (left panel), and number of entries into the open arm (right panel). Data represent mean ± SEM. Data in (C, E) from 10 APP/PS1 mice and 15 AQP4*^-/-^*/APP/PS1 mice were analyzed by Student’s t-test. Data in (F, G) from 25 mice were analyzed by Pearson correlation analysis.

Conversely, the expression levels of AQP4, a functional protein of glymphatic transport [13-15], was significantly higher in HA-APP/PS1 mice than NA-APP/PS1 mice and WT mice (F_2, 12_ = 14.49, *p* = 0.0006, NA *vs.* WT, *p* = 0.0083; HA *vs*. WT, *p* < 0.0001; HA *vs.* NA, *p* = 0.0388; n = 5 per group, one-way ANOVA with Tukey’s post hoc test; Fig. 5A, B). Consistently, GFAP expression levels were also increased in HA-APP/PS1 mice (F_2, 12_ = 21.17, *p* = 0.0001, NA *vs.* WT, *p* = 0.2624; HA *vs.* WT, *p* = 0.0005; HA *vs.* NA, *p* = 0.0093; n = 5 per group, one-way ANOVA with Tukey’s post hoc test; Fig. 5A, C). We further examined astrocyte activation and AQP4 distribution in the motor cortex by double immunofluorescence staining for GFAP and AQP4. There were only scattered GFAP immunofluorescence in WT mice, but activated GFAP-positive astrocytes characterized by hypertrophic cell bodies and intensely stained processes were observed in APP/PS1 mice [50]. Moreover, activation of astrocytes was further evident in HA-APP/PS1 mice than NA-APP/PS1 mice, as revealed by increases in GFAP positive area percentage (F_2, 9_ = 81.44, *p* < 0.0001, HA *vs.* NA, *p* < 0.0001, n = 4 per group, one-way ANOVA with Tukey’s post hoc test; Fig. 5E). Consistent with the resting state of astrocytes in WT mice, AQP4 immunofluorescence was primarily localized around microvascular and along the pia surface in WT mice. Nevertheless, AQP4 was abnormally localized in the non-vascular regions of APP/PS1 mice, suggesting that its polarity was damaged (Fig. 5D). Quantitative analysis further confirmed that AQP4 polarity, the ratio of perivascular or sub-pial AQP4 immunofluorescence intensity to non-vascular AQP4 immunofluorescence intensity, was significantly lower in HA-APP/PS1 mice than NA-APP/PS1 mice (F_2, 14_ = 68.96, *p* < 0.0001, HA *vs.* NA, *p* = 0.0018, n = 6 per group; F_2, 9_ = 55.53, *p* < 0.0001, HA *vs.* NA, *p* = 0.0062, n = 4 per group, respectively, one-way ANOVA with Tukey’s post hoc test; Fig. 5F, G).

### Evident impaired glymphatic transport in APP/PS1 mice with hyperactivity

Previous studies have shown that AQP4 mislocalization impairs glymphatic transport in several AD mouse models, including APP/PS1 mice [19-22]. We further determined the relationship between glymphatic dysfunction and hyperactivity in APP/PS1 mice. The fluorescence tracer TR-d3 was injected into the frontal motor cortex, and brains were harvested 45 min later. Parenchymal distribution of tracer was evaluated in serial coronal sections of the frontal cortex. The results demonstrated that TR-d3 clearance from the brain was reduced in APP/PS1 mice, especially in those with high locomotor activity (Fig. 2B). Quantitative data further revealed a high percentage of fluorescent areas within the cortex and adjacent brain regions of HA-APP/PS1 mice at different distances from Bregma, compared with that in NA-APP/PS1 mice (bregma 1.18 mm: F_2, 10_ = 63.30, *p* < 0.0001, *p* = 0.0001; bregma 0.38 mm: F_2, 10_ = 24.02, *p* = 0.0002, *p* = 0.0387; bregma -0.58 mm: F_2, 10_ = 20.91, *p* = 0.0003, *p* = 0.0038; bregma -1.22 mm: F_2, 10_ = 24.27, *p* = 0.0001, *p* = 0.0009; bregma -1.82 mm: F_2, 10_ = 25.61, *p* < 0.0001, *p* = 0.0076; WT, n = 5; NA, n = 4; HA, n = 4; repeated measures ANOVA with Tukey’s post hoc test; Fig. 2D). These results were consistent with a lower clearance ability of TR-d3 in HA-APP/PS1 mice (F_2, 10_ = 135.4, *p* < 0.0001, HA *vs.* NA, *p* < 0.0001, WT, n = 5; NA, n = 4; HA, n = 4, one-way ANOVA with Tukey’s post hoc test; Fig. 2E). HA-APP/PS1 mice also showed a lower percentage of TR-d3 fluorescent areas within the dcLNs than NA-APP/PS1 mice (F_2, 10_ = 38.94, *p* < 0.0001, HA *vs.* NA, *p* = 0.0224; Fig. 2G, H). This result was consistent with our previous reports that impaired glymphatic transport hampers clearance of interstitial macromolecules to the dcLNs via meningeal lymphatic vessels [18, 20, 24]. Moreover, relative locomotor activity of mice during wheel testing had a partial negative relationship to their ability to clear injected TR-d3 (*p* = 0.0001, *r* = -0.9645, n = 8, Pearson correlation analysis; Fig. 2F). Altogether, these results suggest that the hyperactivity of APP/PS1 mice may be associated with AQP4 mislocalization and impaired glymphatic transport.

### AQP4 knockout increased hyperactivity of APP/PS1 mice

To further clarify the pathophysiological role of AQP4 in motor over-activity of APP/PS1 mice, we compared the behavioral performance of 4-month-old AQP4*^-/-^*/APP/PS1 mice and APP/PS1 mice in the Y-maze and EPM tests (Fig. 6A). Time spent in, and the number of entrances into, the novel arm of Y maze was comparable between the two-genotypes (*p* = 0.0529, *p* = 0.2744, respectively, APP/PS1, n = 10; AQP4*^-/-^*/APP/PS1, n = 15, Student’s t-test; Fig. 6B, C), but the total distance traveled by AQP4*^-/-^*/APP/PS1 mice was significantly higher than that of APP/PS1 mice (*p* = 0.0025, APP/PS1, n = 10; AQP4*^-/-^*/APP/PS1, n = 15, Student’s t-test; Fig. 6B, C). AQP4*^-/-^*/APP/PS1 mice also displayed hyperactivity during the EPM test, without alterations in the open arm time spent and entering numbers, compared to APP/PS1 mice (time spent, *p* = 0.1675; entering number, *p* = 0.1562; distance traveled, *p* = 0.0021, APP/PS1, n = 10; AQP4*^-/-^*/APP/PS1, n = 15, Student’s t-test; Fig. 6D, E). The movement distance in the Y-maze was not correlated with the time spent in the new arm or number of entrances into the new arm (APP/PS1 mice plus AQP4*^-/-^*/APP/PS1 mice: *p* = 0.3945, *r* = 0.1781; *p* = 0.6162, *r* = 0.1054, respectively, n = 25; AQP4*^-/-^*/APP/PS1 mice: *p* = 0.9959, *r* = -0.00146; *p* = 0.4858, *r* = 0.1951, respectively, n = 15, Pearson correlation analysis; Fig. 6F, Supplementary Fig. 5A). Similarly, their movement distance in the EPM did not correlate with residence time or entry times in the open arm (APP/PS1 mice plus AQP4*^-/-^*/APP/PS1 mice: *p* = 0.2869, *r* = 0.2217; *p* = 0.1706, *r* = 0.2829, respectively, n = 25; AQP4*^-/-^*/APP/PS1 mice: *p* = 0.2612, *r* = 0.3097; *p* = 0.5931, *r* = 0.1502, respectively, n = 15, Pearson correlation analysis; Fig. 6G, Supplementary Fig. 5B). These results indicate that AQP4 absence in APP/PS1 mice does not exacerbate memory decline or anxiety-like phenotype but causes hyperactivity in the early stage of AD-like pathology.

**Figure 7.**
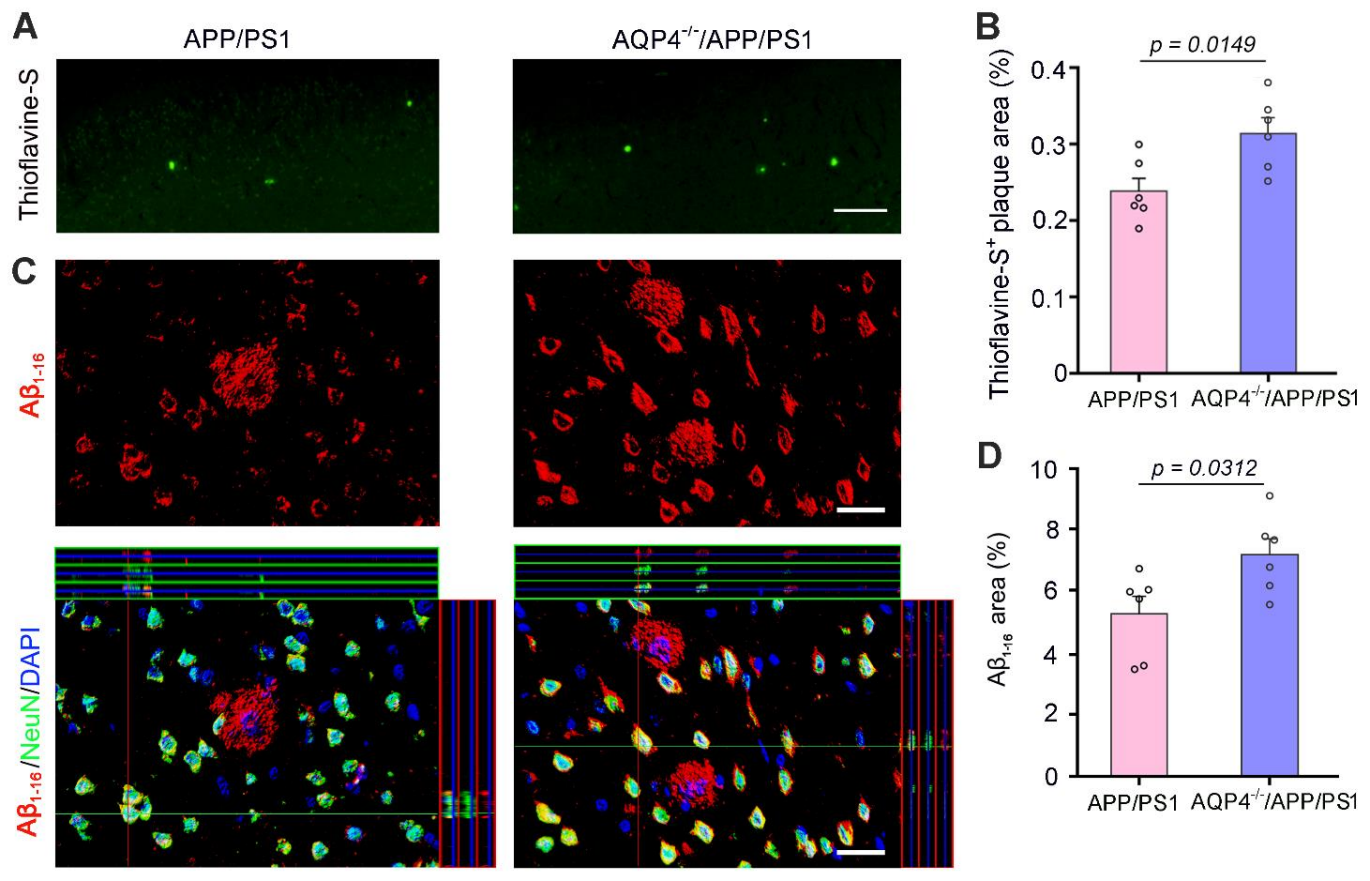
**AQP4 deletion increased Aβ load in the frontal cortex of 4-month-old APP/PS1 mice**. (**A**) Representative images show Thioflavine-S positive plaques in the frontal cortex of APP/PS1 mice and AQP4*^-/-^*/APP/PS1 mice. Scale bar, 200 μm. (**B**) The percentage of Thioflavine-S positive plaque area of the two-genotype mice. (**C**) Representative images of Aβ_1-16_ immunofluorescence revealed that Aβ plaques and intraneuronal Aβ were more evident in AQP4*^-/-^*/APP/PS1 mice than APP/PS1 controls. Scale bar, 15 μm. (**D**) The percentage of Aβ_1-16_ positive plaque areas of the two-genotype mice. Data represent mean ± SEM of 3 sections per mouse with 6 mice per group. Data were analyzed by Student’s t-test.

### Increased Aβ accumulation and neuronal activation in AQP4^-/-^/APP/PS1 mice

Thioflavine-S positive Aβ plaques increased in AQP4^-/-^/APP/PS1 mice (*p* = 0.0149, n = 6 per group Student’s t-test; Fig. 7A, B). NeuN and Aβ_1-16_ double immunofluorescence also showed that both intraneuronal Aβ accumulation and extracellular Aβ plaque deposition were more significant in AQP4^-/-^/APP/PS1 mice than APP/PS1 mice (*p* = 0.0312, n = 6 per group, Student’s t-test; Fig. 7C, D). Meanwhile, protein expression levels of Aβ production and clearance related indexes including APP, ADAM10, BACE1, PS1, NEP and IDE were not different between AQP4*^-/-^*/APP/PS1 mice and APP/PS1 mice (*p* = 0.0754, *p* = 0.9160, *p* = 0.5377, *p* = 0.0537, *p* = 0.4812, *p* = 0.4739, respectively, n = 4 per group, Student’s t-test; Supplementary Fig. 6A, B)

**Figure 8.**
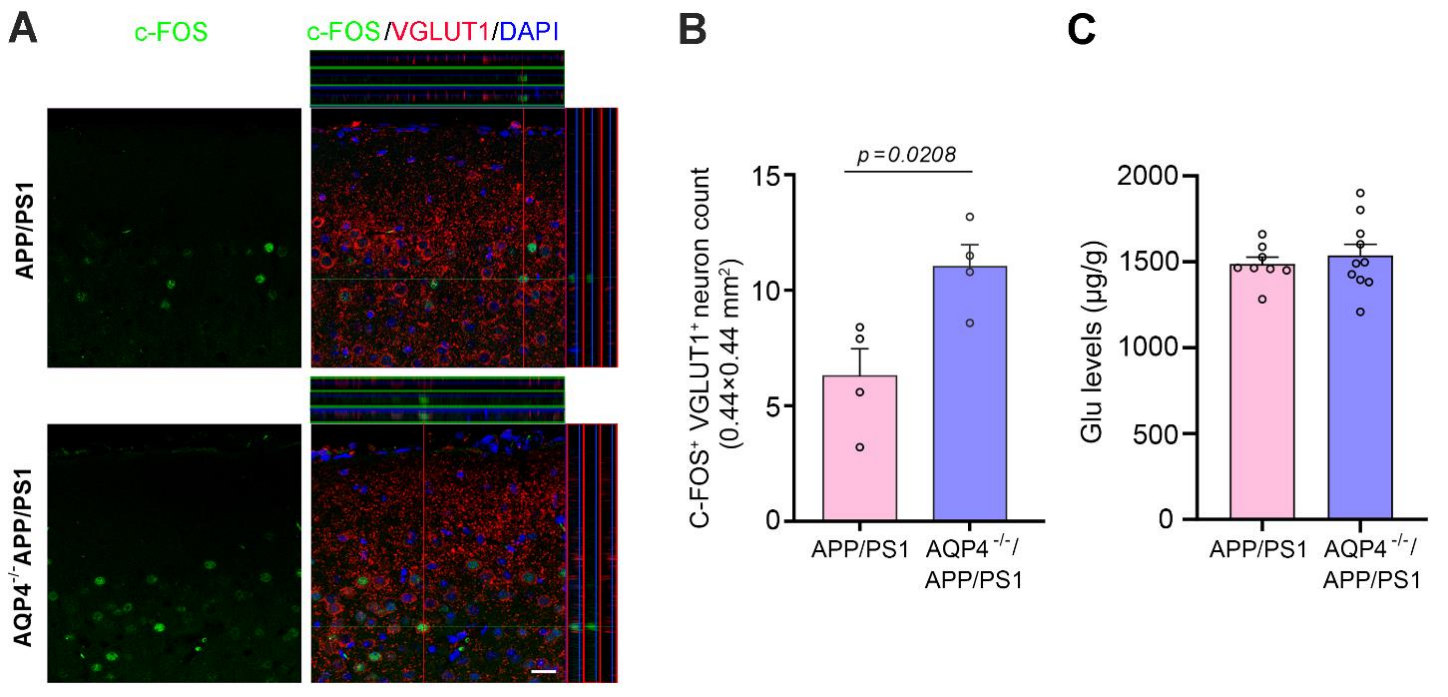
**AQP4 deletion increased activation of glutamatergic neurons in the motor cortex of 4-month-old APP/PS1 mice**. (**A**) Representative images show that the proportion of VGluT1 positive neurons expressing c-Fos was higher in AQP4*^-/-^*/APP/PS1 mice than APP/PS1 mice. Scale bar, 15 μm. (**B**) Quantification of c-Fos and VGLUT1 double labeled neurons per high magnification field (0.44 mm × 0.44 mm) of the two-genotype mice. (**C**) Quantification of Glu concentrations measured by HPLC. Data represent mean ± SEM. Data in (B) from 3 sections per mouse with 4 mice per group and in (C) from triplicate independent experiments with 8 APP/PS1 mice and 10 AQP4*^-/-^*/APP/PS1 mice were analyzed by Student’s t-test.

We also compared the activation of glutamatergic neurons in the motor cortex between the two genotypes and found that the number of c-Fos and VGLUT1 double labeled neurons was increased significantly in the frontal cortex of AQP4*^-/-^*/APP/PS1 mice (*p* = 0.0208, n = 4 per group, Student’s t-test; Fig. 8A, B). HPLC analysis of frontal cortex homogenate samples showed that GABA levels were mildly increased in AQP4*^-/-^*/APP/PS1 mice *(p* = 0.0469, APP/PS1, n = 8; AQP4*^-/-^*/APP/PS1, n = 10, Student’s t-test), but concentrations of other amino acid neurotransmitters were not different between AQP4*^-/-^*/APP/PS1 mice and APP/PS1 mice (Glu: *p* = 0.5525, Asp: *p* = 0.1907, Ser: *p* = 0.2304, Gln: *p* = 0.2340, Gly: *p* = 0.2065, Tau: *p* = 0.2825, *p* = 0.0469, respectively, APP/PS1, n = 8; AQP4*^-/-^*/APP/PS1, n = 10, Student’s t-test; Fig. 8C, Supplementary Fig. 7). The above results indicate that AQP4 deficiency increases Aβ accumulation and causes motor neuronal hyperactivity, subsequently exhibiting hyperactivity.

## DISCUSSION

BPSD is a common clinical symptom of AD, which exists in more than 90% of AD patients [51, 52], and can occur at almost any stage of AD. In some AD cases, these symptoms may even appear before mild cognitive impairment [53, 54]. Among them, hyperactivity-impulsivity-irritiability-disinhibition-aggression-agitation (HIDA) is one of the most difficult aspects of AD treatment, which exacerbates the burden on caregivers and hospital staff [55]. In particular, wandering behavior, characterized by aimless movement, may be one of the main clinical manifestations in the preclinical or prodromal stage of AD, increasing the risk of patient falls and/or walking away from their home [56]. Therefore, exploring the underlying mechanism of HIDA is of great significance for the early diagnosis and intervention of AD.

The abnormal neural circuit and pathological basis of HIDA are unclear, but may be related to disinhibition of the cortical-striatal circuit because of Aβ-induced neuronal hyperexcitation [39, 40]. In agreement with this view, the present results demonstrated there are increases in intraneuronal accumulation of Aβ and expression of c-Fos in glutamatergic neurons of the motor cortex of hyperactive APP/PS1 mice compared to those in APP/PS1 mice with normal motor activity. It is well known that intraneuronal accumulation of Aβ is involved in synaptic dysfunction, cognitive impairment, and formation of amyloid plaques in AD [57, 58]. Moreover, soluble Aβ results in neuronal hyperactivity, which, in turn triggers soluble Aβ production, thus forming a vicious cycle that promotes the onset of AD [59-61]. In this regard, addressing the key mechanism leading to an initial accumulation of Aβ in the motor control regions will be beneficial for early prevention and intervention of HIDA occurrence in prodromal AD patients.

It is believed that Aβ accumulation is caused by an imbalance between its production and clearance [48]. Impairments of Aβ clearance systems, such as enzymatic degradation, cellular uptake, and transport across the blood-brain barrier, are involved in excessive Aβ deposits in sporadic AD [49]. Among them, glymphatic dysfunction could be a main contributor to Aβ accumulation, as revealed by recent evidence from animal experiments [16-17, 19-24] as well as clinical observations [62-64]. Indeed, the brain has the most abundant blood supply in the entire body. There is a potential and deformable space between these tens of thousands of microvessels and adjacent brain parenchyma, providing a huge interface for the exchange between cerebrospinal fluid and interstitial fluid. This is conducive to the removal of various brain metabolites, thus playing a crucial role in maintaining brain homeostasis [49].

As mentioned above, the paravascular space is surrounded by astrocyte endfeet which containing vast amounts of AQP4 [13-15]. This unique molecular anatomical structure not only promotes rapid water transport from the interstitial space across the astrocyte membrane to the paravascular space, but also causes corresponding plastic changes in the gap between astrocyte processes. Consequently, it promotes removal of macromolecular metabolites, including Aβ, from the brain parenchyma to the perivenous space [14]. Therefore, this clearance efficiency depends on the perivascular polarization of AQP4. However, under various pathological conditions, astrocytes are activated with abnormal expression of AQP4 on its non-vascular membrane domains, subsequently impairing AQP4 polarization and glymphatic clearance function [16-20]. In the present study, we found that astrocyte activation and mislocalization of AQP4 in hyperactive APP/PS1 mice were distinct when compared with those in APP/PS1 mice with normal activity. AQP4 deletion in 4-month-old APP/PS1 mice increased Aβ load, further suggesting that an impairment of AQP4 mediated glymphatic clearance plays a key role in Aβ pathogenesis.

Apart from regulation of Aβ transport, AQP4 has also been implicated in the metabolism and release of amino acids [65-68]. For examples, despite no changes in the baseline levels of Glu and GABA in the frontal cortex, when exposed to ketamine AQP4^-/-^ mice showed lower Glu levels and higher GABA levels compared with WT mice [66]. Deletion of AQP4 also reduces extracellular Glu elevation in high KCl-induced cortical spreading depression [67]. In the present study, we revealed that activation of glutamatergic neurons was more evident in the frontal cortex of AQP4^-/-^/APP/PS1 mice than APP/PS1 mice, but Glu levels were comparable between the two genotypes. One possible reason is that AQP4 deficiency expands extracellular space volume, subsequently hampering an elevation in Glu concentrations in response to various stimuli [13, 67]. The exact mechanisms responsible for these alterations are still unclear and warrant further investigation.

Currently, most fundamental research on AD transgenic mice for HIDA-like symptoms focuses primarily on impulsive behavior, aggression, and mania [29-35]. There are few reports on hyperactivity, and the results are inconsistent. For example, in a previous study 12-month-old APP/PS1 mice displayed an increased movement distance in the central area of the open field compared with the control mice [69]. One study reported that APP/PS1 mice at 7 months old exhibited an excessive movement phenotype in the light and dark box but had normal activity performance in the EPM [70]. However, another study reported that motor activity of APP/PS1 mice at 9 months decreased during open field testing [71]. According to a recent study, the total distance traveled, and distance in the central area in the open field, were similar between 12-month-old APP/PS1 mice and age-matched WT controls. In the Y-maze, overall activity of these APP/PS1 mice was normal but showed significant individual differences [72]. The present study confirmed that a considerable portion of APP/PS1 mice showed hyperactivity both in the Y-maze, EPM and open field tests in the early stages of AD-like processes. An early study from our group reported that 16-week-old APP/PS1 mice spent less time in the central area of the open field than WT mice, although the numbers of entries into the central area were similar. These APP/PS1 mice also exhibited high locomotor hyperactivity, which was not significantly impaired after chronic mild stress for 8 weeks [26]. The varying results from the above observations may be related to different methods and time points of detection.

BPSD tends to be more severe at night than during the day, which is mainly due to damage of circadian rhythm in AD patients [73]. As in humans, motor activity of mice is also regulated by circadian rhythm [74]. Behavioral testing in our study was conducted during the daytime, which is equivalent to the resting time of mice. It is worth exploring whether hyperactivity of APP/PS1 mice also has a similar circadian rhythm dependent pattern. Recent studies have discovered that AQP4 mediated glymphatic clearance is under circadian control. Perivascular polarization of AQP4 is highest during the resting phase, and deletion of AQP4 eliminates the day-night variance in glymphatic transport in mice [75]. Therefore, it is necessary to further investigate whether circadian variation of AQP4 polarity is altered in the early pathologic stage of APP/PS1 mice.

In this study we found hyperactivity with significant inter-individual variability in 4-month-old APP/PS1 mice. Our results highly indicate that AQP4 mislocalization or deficiency leads to increased intraneuronal Aβ load and neuronal hyperactivity in the motor cortex, which in turn causes locomotor over-activity during the early pathophysiology of APP/PS1 mice. However, the exact mechanism of individual differences in glymphatic transport of APP/SP1 mice has not been addressed in the current study. Actually, the phenotypes of AD Transgenic mice, including APP/SP1 and 5xTg mice, showed significant individual differences. For instance, Gureviciene et al. (2019) reported different physiological activities among APP/PS1 mice [76]. Some electroencephalogram spikes, such as spike-wave discharges, cortico-hippocampal spikes with after hyperpolarization and giant spikes, could only be detected from brains of a subset of APP/PS1 mice. Many factors, including Aß, connexins and cytokines, can affect AQP4 expression levels and polarity [17, 77, 78]. For example, an interesting paper recently reported that brain interleukin33 is required for regulation of AQP4 expression in astrocytes, especially those at neuron-facing membrane domain [17]. Therefore, further work needs to be explored the individual difference of glymphatic transport impairment in AD model mice.

Consistent with animal experiment results, alterations in perivascular polarization of AQP4 are observed in the fronto-temporal lobe of AD patients, with the degree of disease progression related to single nucleotide polymorphisms (SNPs) of AQP4 [64, 79]. AQP4 SNPS are also involved in the occurrence of the temporal lobe epilepsy [80], as well as schizophrenia [81]. A recent study reported that functional variants of AQP4 modulate deep non-rapid eye movement sleep and cognitive consequences of prolonged wakefulness [82]. These clues indicate an involvement of AQP4 SNPS in the occurrence of HIDA of patients with AD. Further research is needed to confirm this presumption.

In summary, the present study revealed that a proportion of APP/PS1 mice have hyperactive motor performance during early-stage AD-like pathology. This abnormal phenotype is associated with hyperexcitability of cortical neurons, which may be due to accumulation of intraneuronal Aβ caused by mislocalization of the perivascular pool of AQP4. Protecting AQP4 polarization may offer a new venue for early intervention of hyperactivity in patients with AD.

## Supplementary Materials

The Supplementary data can be found online at: www.aginganddisease.org/EN/10.14336/AD.2021.0627.


