# Supplementary material for: Locomotor Hyperactivity in the Early-Stage Alzheimer’s Disease-like Pathology of APP/PS1 Mice: Associated with Impaired Polarization of Astrocyte Aquaporin 4
Source: Aging Dis. 2022 Oct 1;13(5):1504–22. doi: 10.14336/AD.2022.0219 (PMC9466968; doi:10.14336/AD.2022.0219)
Supplement: Supplementary file 1 [file AD-13-5-1504-s.pdf]

SUPPLEMENTARY DATA

Locomotor Hyperactivity in the Early-Stage Alzheimer's Disease-like Pathology of APP/PS1 Mice: Associated with Impaired Polarization of Astrocyte Aquaporin 4

Tianqi Wang[1,2,#], Yan Chen[1,2,#], Ying Zou[1], Yingting Pang[1], Xiaoxin He[1,2], Yali Chen[1,3], Yun Liu[1], Weixi Feng[1,2], Yanli Zhang[1], Qian Li[1,2], JingpingShi[4], Fengfei Ding[5], Charles Marshall[6], Junying Gao[1*], Ming Xiao[1,2*]

© 2022. Wang T. et al. Published online at http://www.aginganddisease.org/EN/10.14336/AD.2022.0218

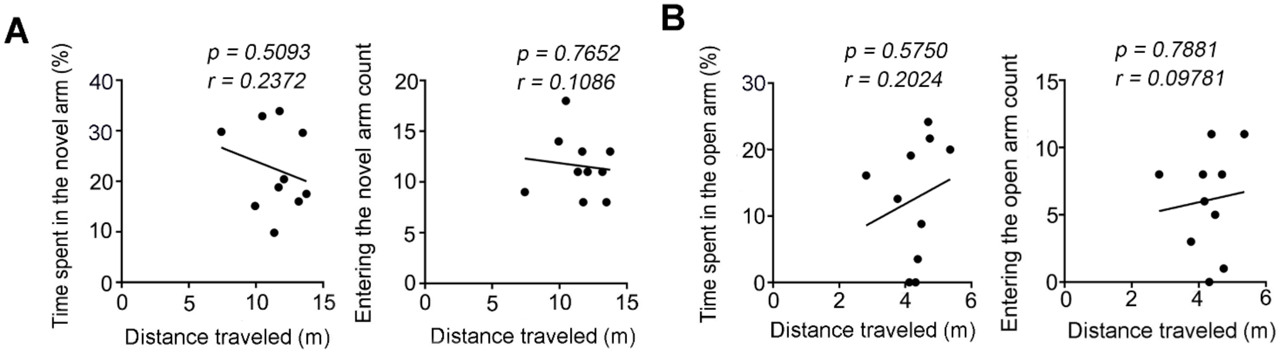

Supplementary Figure 1. Correlation analysis of distance traveled and anxiety related indicators of hyper-active APP/PS1 mice. (**A**) Correlation analysis of total distance traveled during the Y-maze test and percentage of time spent in the novel arm (left panel), and number of entries into the novel arm (right panel), respectively. (**B**) Correlation analysis of total distance traveled during the EMP test and percentage of time spent in the open arm (left panel), and number of entries into the open arm (right panel), respectively. Data from 10 hyper-active APP/PS1 mice were analyzed by Pearson correlation analysis.

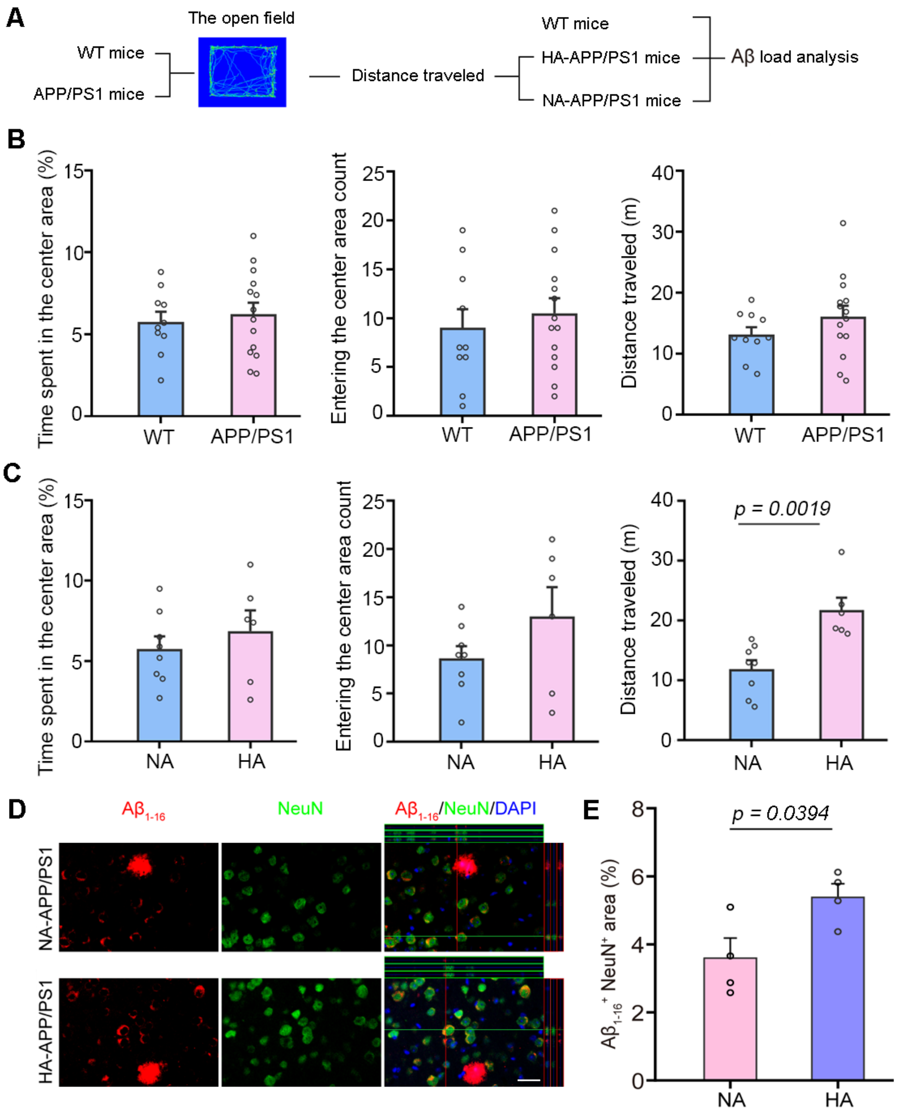

© 2022. Wang T. et al. Published online at http://www.aginganddisease.org/EN/10.14336/AD.2022.0218

Supplementary Figure 2. Increased Aβ load in APP/PS1 mice with hyperactivity. (**A**) Schematic diagram of the experimental procedure showing that WT mice and APP/PS1 mice received the open field test followed by Aβ load analyses. (**B**) Percentage of time spent in the central area (left panel), number of entries into the central area (middle panel), and total distance traveled (right panel) of APP/PS1 mice and WT mice in the open field test. (**C**) The percentage of time spent in the central area (left panel) and number of entries into the central area (middle panel) were not different between APP/PS1 mice with long distance and those with normal locomotor activity (right panel). (**D**) Representative images of $A\beta_{1-16}$ in HA-APP/PS1 mice and NA-APP/PS1 mice. Scale bar, 30 µm. (**E**) HA-APP/PS1 mice exhibited a higher percentage of $A\beta_{1-16}$ positive area than NA-APP/PS1 mice. Data represent mean ± SEM. Data in (**B**) from 10 WT mice and 14 APP/PS1 mice, in (**C**) from 6HA-APP/PS1 mice and 8 NA-APP/PS1 mice, and in (**E**) from 3 sections per mouse with 4 mice per group were analyzed by Student's t-test.

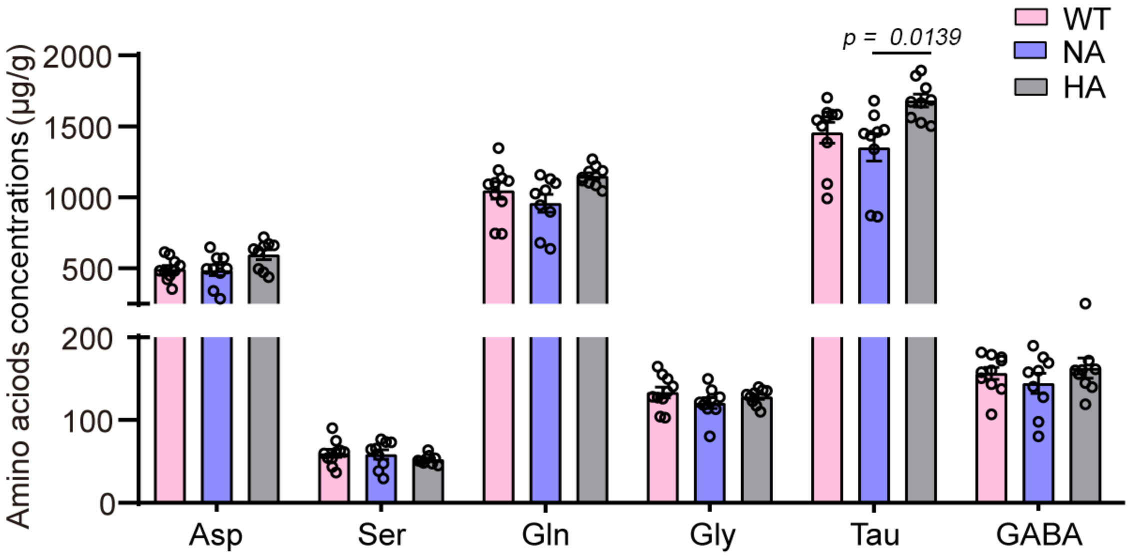

Supplementary Figure 3. HPLC analysis of the concentrations of amino acid neurotransmitters in the frontal cortex of 4-month-old WT mice, and APP/PS1 mice with hyperactivity (HA) and normal activity (NA). Quantification of Asp, Ser, Gln, Gly, Tau and GABA concentrations. Data represent mean ± SEM of triplicate independent experiments with 10 WT mice, 9HA-APP mice and 9 NA-APP/PS1 mice. Data were analyzed one-way ANOVA with Tukey's post hoc test.

© 2022. Wang T. et al. Published online at http://www.aginganddisease.org/EN/10.14336/AD.2022.0218

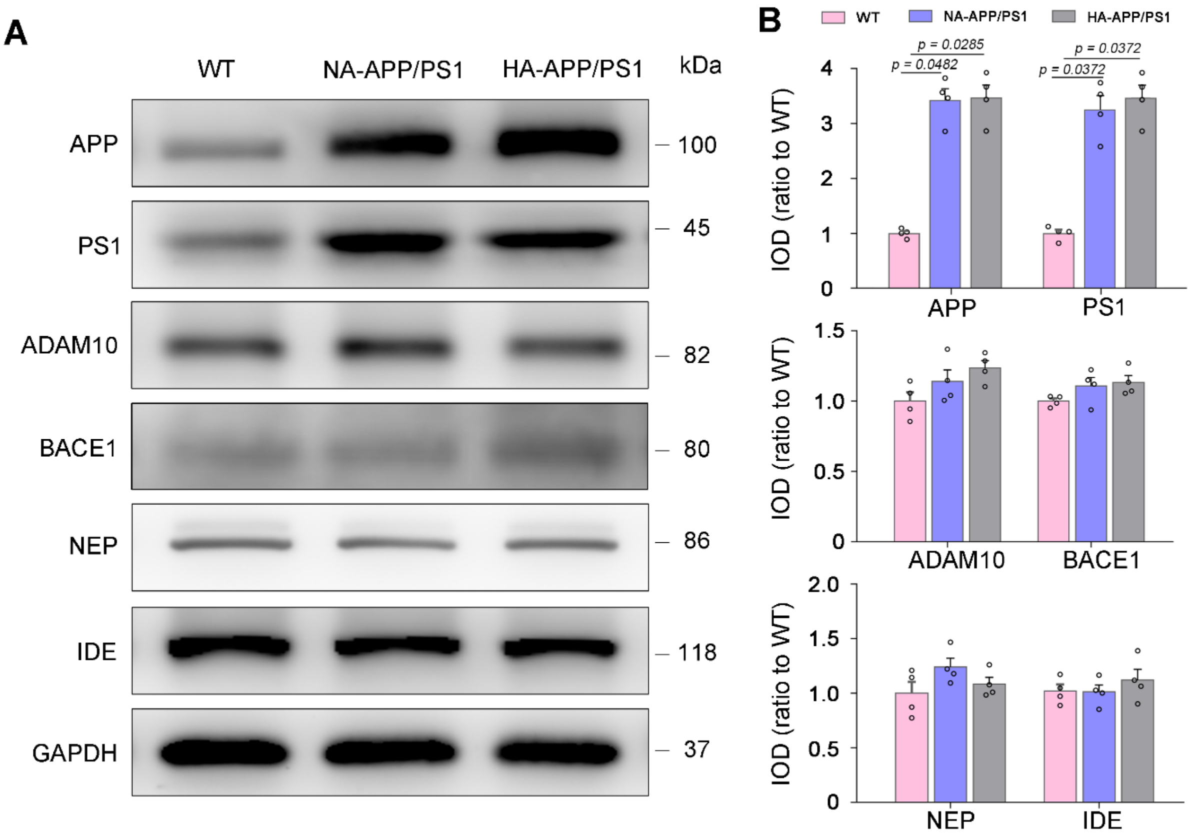

Supplementary Figure 4. Analysis of Aβ metabolism-related markers in the frontal cortex of 4-month-old mice and APP/PS1 mice with hyperactivity and normal activity. (**A, B**) Representative Western blot and densitometric analysis of APP, PS1, ADAM10, BACE1, NEP and IDE in the frontal cortex of WT mice, hyperactivity (HA)-APP/PS1 mice and normal activity (NA)-APP/PS1 mice. The integrated optical density (IOD) was shown as a relative expression ratio to the WT level. Data represent mean ± SEM of duplicate independent experiments with 4 mice per group. Data for APP and PS1 levels were analyzed by Kruskal-Wallis test, for ADAM10, BACE1, NEP and IDE levels were analyzed by one-way ANOVA with Tukey's post hoc test.

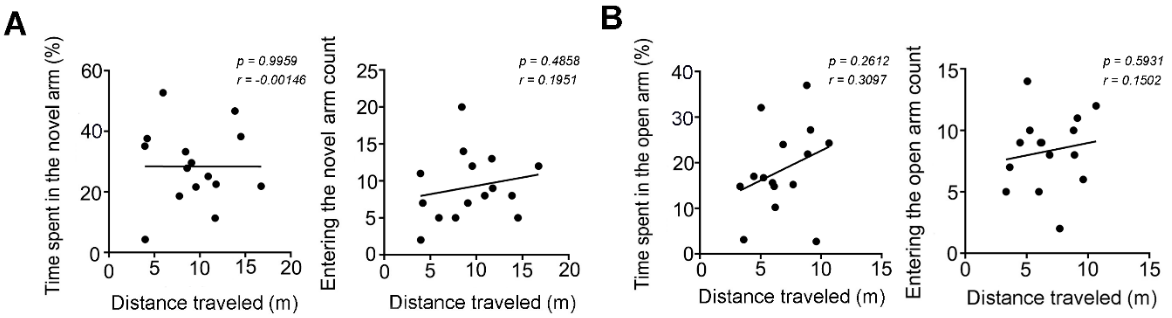

Supplementary Figure 5. Correlation analysis of distance traveled and anxiety related indicators of AQP4$^{-/-}$/APP/PS1 mice. (**A**) Correlation analysis of total distance traveled during the Y-maze test and percentage of time spent in the novel arm (left panel), and number of entries into the novel arm (right panel), respectively. (**B**) Correlation analysis of total distance traveled during the EMP test and percentage of time spent in the open arm (left panel), and number of entries into the open arm (right panel), respectively. Data from 15 AQP4$^{-/-}$/APP/PS1 mice were analyzed by Pearson correlation analysis.

© 2022. Wang T. et al. Published online at http://www.aginganddisease.org/EN/10.14336/AD.2022.0218

SUPPLEMENTARY DATA

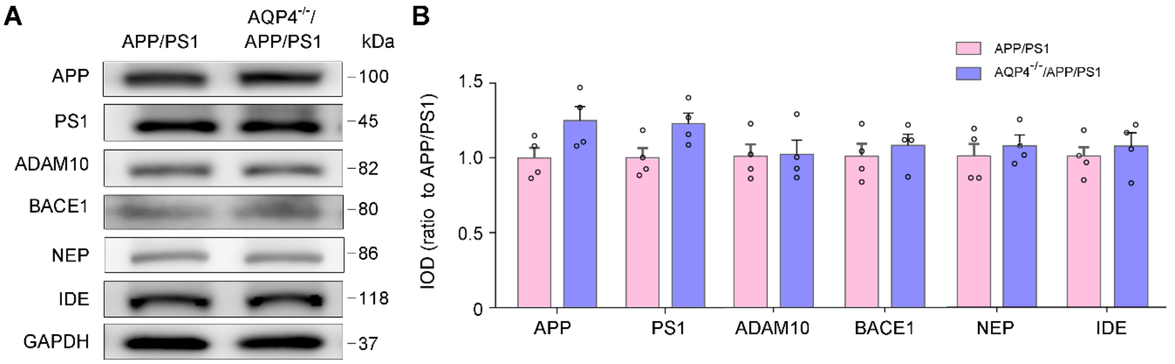

Supplementary Figure 6. AQP4 deletion did not affect expression levels of Aβ metabolism-related markers of 4-month-old APP/PS1 mice. (**A, B**) Representative Western blot and densitometric analysis of APP, PS1, ADAM10, BACE1, NEP and IDE in the cerebral cortex of APP/PS1 mice and AQP4$^{-/-}$/APP/PS1. The integrated optical density (IOD) was shown as a relative expression ratio to the WT level. Data represent mean ± SEM of duplicate independent experiments with 4 mice per group. Data were analyzed by Student's t-test.

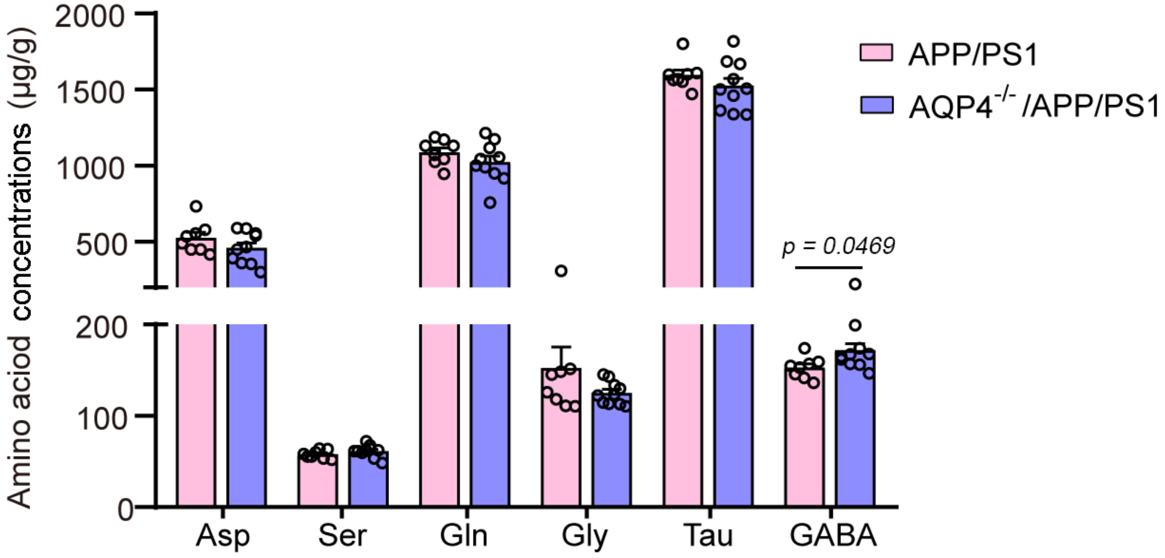

Supplementary Figure 7. HPLC analysis of the concentrations of amino acid neurotransmitters in the frontal cortex in the frontal cortex of 4-month-old APP/PS1 mice and AQP4$^{-/-}$/APP/PS1 mice. Quantification of Asp, Ser, Gln, Gly, Tau and GABA concentrations. Data represent mean ± SEM of triplicate independent experiments with 8 APP/PS1 mice and 10 AQP4$^{-/-}$/APP/PS1 mice. Data were analyzed the one-way ANOVA with Tukey's post hoc test.

© 2022. Wang T. et al. Published online at http://www.aginganddisease.org/EN/10.14336/AD.2022.0218
